# Enhanced protective immunity against SARS-CoV-2 elicited by a VSV vector expressing a chimeric spike protein

**DOI:** 10.1038/s41392-021-00797-9

**Published:** 2021-11-10

**Authors:** Hongyue Li, Yuhang Zhang, Dong Li, Yong-Qiang Deng, Hongde Xu, Chaoyue Zhao, Jiandong Liu, Dan Wen, Jianguo Zhao, Yongchun Li, Yong Wu, Shujun Liu, Jiankai Liu, Junfeng Hao, Fei Yuan, Shuguang Duo, Cheng-Feng Qin, Aihua Zheng

**Affiliations:** 1 State Key Laboratory of Integrated Management of Pest Insects and Rodents, Institute of Zoology, Chinese Academy of Sciences, 100101 Beijing, China; 2 CAS Center for Excellence in Biotic Interactions, University of Chinese Academy of Sciences, 100101 Beijing, China; 3 Shenzhen Kangtai, Biotechnology Co., Ltd, 518106 Shenzhen, Guangdong China; 4 State Key Laboratory of Pathogen and Biosecurity, Institute of Microbiology and Epidemiology, Academy of Military Medical Sciences, 100071 Beijing, China; 5 School of Pharmaceutical Sciences, Zhengzhou University, 450001 Zhengzhou, Henan China; 6 State Key Laboratory of Stem cell and Reproductive Biology, Institute of Zoology, Chinese Academy of Sciences, 100101 Beijing, China; 7 Division of Animal Model Research, Institute for Laboratory Animal Resources, National Institutes for Food and Drug Control, 102629 Beijing, China; 8 Laboratory Animal Center, Institute of Zoology, Chinese Academy of Sciences, 100101 Beijing, China; 9 Core Facility for Protein Research, Institute of Biophysics, Chinese Academy of Sciences, 100101 Beijing, China; 10 College of Life Science, Henan Normal University, 453007 Xinxiang, China

**Keywords:** Vaccines, Vaccines

## Abstract

SARS-CoV-2 and SARS-CoV are genetically related coronavirus and share the same cellular receptor ACE2. By replacing the VSV glycoprotein with the spikes (S) of SARS-CoV-2 and SARS-CoV, we generated two replication-competent recombinant viruses, rVSV-SARS-CoV-2 and rVSV-SARS-CoV. Using wild-type and human ACE2 (hACE2) knock-in mouse models, we found a single dose of rVSV-SARS-CoV could elicit strong humoral immune response via both intranasal (i.n.) and intramuscular (i.m.) routes. Despite the high genetic similarity between SARS-CoV-2 and SARS-CoV, no obvious cross-neutralizing activity was observed in the immunized mice sera. In macaques, neutralizing antibody (NAb) titers induced by one i.n. dose of rVSV-SARS-CoV-2 were eight-fold higher than those by a single i.m. dose. Thus, our data indicates that rVSV-SARS-CoV-2 might be suitable for i.n. administration instead of the traditional i.m. immunization in human. Because rVSV-SARS-CoV elicited significantly stronger NAb responses than rVSV-SARS-CoV-2 in a route-independent manner, we generated a chimeric antigen by replacing the receptor binding domain (RBD) of SARS-CoV S with that from the SARS-CoV-2. rVSV expressing the chimera (rVSV-SARS-CoV/2-RBD) induced significantly increased NAbs against SARS-CoV-2 in mice and macaques than rVSV-SARS-CoV-2, with a safe Th1-biased response. Serum immunized with rVSV-SARS-CoV/2-RBD showed no cross-reactivity with SARS-CoV. hACE2 mice receiving a single i.m. dose of either rVSV-SARS-CoV-2 or rVSV-SARS-CoV/2-RBD were fully protected against SARS-CoV-2 challenge without obvious lesions in the lungs. Our results suggest that transplantation of SARS-CoV-2 RBD into the S protein of SARS-CoV might be a promising antigen design for COVID-19 vaccines.

## Introduction

Severe acute respiratory syndrome coronavirus 2 (SARS-CoV-2) is single-stranded positive sense enveloped RNA virus.^1–3^ After its initial outbreak in December 2019, SARS-CoV-2 has infected more than 175 million humans worldwide and caused more than 3.8 million deaths (as of June 2021, covid19.who.it). Currently, several COVID-19 vaccines, including mRNA vaccines, adenovirus-based vaccines, subunit vaccine, and inactivated vaccines have been approved for emergency use (www.who.int). However, the duration of protection and the long-term side effects are unknown. Thus, comprehensive studies of vaccines are needed to limit virus spread and end the COVID-19 pandemic.

The SARS-CoV-2 spike protein (S) is responsible for receptor binding and membrane fusion. The S protein is composed of two subunits, S1 and S2, which are cleaved by furin protease.^4^ S1 binds with high affinity to the host receptor angiotensin-converting enzyme 2 (ACE2).^5^ S2 forms a hairpin structure to trigger viral–host membrane fusion. The S protein, especially the receptor binding domain (RBD), is the major target of neutralizing antibodies.^6^ Most vaccine platforms, such as adenovirus-vector, recombinant protein subunit and nucleic acid vaccines, use the S or RBD domain as the immunogen.^7–9^ Severe acute respiratory syndrome coronavirus (SARS-CoV), a close relative of SARS-CoV-2,^10,11^ shares 75% identical amino acids in the S protein with SARS-CoV-2. They both use ACE2 as the host receptor^12,13^ and display similar ACE2 binding motifs in the S protein.^14,15^ The distribution and abundance of ACE2 in organs is related to the clinical symptoms of COVID-19.^16^ ACE2 is broadly expressed in heart, kidneys, testes, lungs, liver, intestine, and brain. Furthermore, ACE2 is present in arterial and venous endothelial cells and arterial smooth muscle cells in almost all organs, with low level of ACE2 in the skeletal muscle.^17^

Vesicular stomatitis virus (VSV) is single-stranded, negative-sense RNA virus in the *Rhabdoviridae* family. VSV causes mild symptoms in animals and a few human cases. The serum prevalence of VSV in humans is low. The VSV genome encodes five structural proteins including nucleoprotein (N), phosphoprotein (P), matrix (M), glycoprotein (G), and RNA-dependent polymerase (L). Among these, G is the envelope protein that mediates receptor binding and membrane fusion. VSV can tolerate various heterogeneous viral envelope proteins in place of G to generate recombinant viruses.^18^ Recombinant VSV (rVSV) expressing heterogeneous viral proteins on the surface could mimic the entry process of the target virus and induce specific immune responses. VSV-based Ebola vaccine has been successfully developed and approved for use.^19–21^ Recently, two groups developed replication-competent VSV vaccines expressing the S protein of SARS-CoV-2, which could confer protection in rodents against SARS-CoV-2 challenge.^22,23^

Although SARS-COV-2 is genetically close to SARS-CoV, the two viruses differ in infectivity, pathogenesis, and immune responses.^24^ Clinical surveillances suggest that the humoral immune response of SARS and COVID-19 patients are significantly different. In a cohort of 18 SARS patients, the average NAb titer was 590 at 30 days of post-infection and remained higher than 100 for at least 8 months.^25^ In another cohort study of 56 convalescent patients with SARS, the titers of NAb peaked at 4 months of post-infection and were higher than 1000 for at least 6 months.^26^ A 3-year follow-up study revealed that neutralization antibodies persisted at a titer of around 1000 for at least 16 months after SARS-CoV infection.^27^ These studies demonstrated that SARS-CoV elicit robust and long-lasting humoral immune responses in humans. In contrast, the NAb levels in SARS-CoV-2 convalescent patients was relatively low.^28^ In a cohort of 149 patients, the mean NAb titer was 121, measured using HIV-1 based pseudovirus, with 33% of the samples less than 50.^29^ Similarly, the mean pseudovirus NAb titer was ~200 in another cohort of 188 COVID-19 patients.^30^ Based on a dataset of 30,082 COVID-19 positive individuals screened at Mount Sinai Health System in New York City, the average NAb titer was about 200–300 as determined by authentic SARS-CoV-2.^31^

To increase the efficacy of SARS-CoV-2 vaccines, many efforts have been devoted in the design of antigens. Proline substitution in the S2 domain resulted in high yield of the pro-fusion ectodomain,^32,33^ which could increase the immunogenicity of S protein. This design was adapted by almost all the nucleic acid-based and vector-based COVID-19 vaccines. Dimeric RBD formed by insertion of a cross-monomer disulfide bond, results in significantly increased NAb titers as compared with conventional monomeric form.^34^ In this report, we constructed the replication-competent VSV-vectored recombinant SARS-CoV and SARS-CoV-2 respectively (assigned as rVSV-SARS-CoV and rVSV-SARS-CoV-2). By immunization in mouse and non-human primate animal models, we found rVSV-SARS-CoV could elicit much stronger immune responses than rVSV-SARS-CoV-2. To further enhance the vaccine efficacy, we transplanted the RBD of SARS-CoV-2 S into SARS-CoV S to obtain a new VSV-based vaccine candidate (rVSV-SARS-CoV/2-RBD). A single dose of rVSV-SARS-CoV/2-RBD produced significantly higher NAb responses than rVSV-SARS-CoV-2 in mouse and monkey models and conferred protection in the hACE2 knock-in mouse model. Our research paves the route for further development of a VSV-vectored COVID-19 vaccine.

## Results

### Characterization of rVSV-SARS-CoV-2 and rVSV-SARS-CoV

**Fig. 1:**
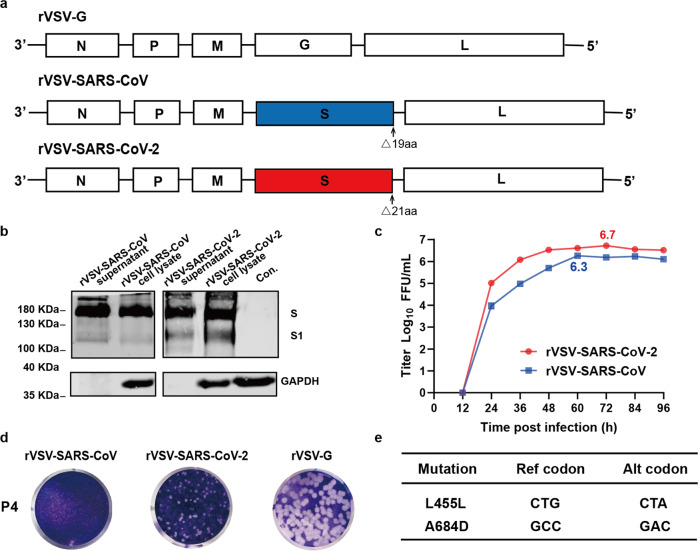
Characterization of rVSV-SARS-CoV and rVSV-SARS-CoV-2. **a** Schematic diagrams showing genome organization of rVSV vectors. The original VSV G of rVSV-G was replaced by the SARS-CoV S lacking 19 aa at the C-terminus to generate rVSV-SARS-CoV. The original VSV G gene was replaced by the SARS-CoV-2 S lacking 21 aa at the C-terminus to generate rVSV-SARS-CoV-2. **b** Purified rVSV-SARS-CoV and rVSV-SARS-CoV-2 from supernatants (Lane 1 and 3), lysates of rVSV -SARS-CoV and rVSV -SARS-CoV-2 infected Vero cells (Lane 2 and 4) and mock-infected Vero cells (Con.) (Lane 5) were blotted with antibodies recognizing the S protein of SARS-CoV or SARS-CoV-2, using GAPDH as input control. **c** Growth kinetics of rVSV-SARS-CoV and rVSV-SARS-CoV-2. Vero cells were infected by the rVSV viruses (MOI = 0.01) and virus titers in the supernatant were measured at the indicated time points post infection by immunofluorescence on Vero cells in duplicate. **d** Plaque morphology of rVSVs in Vero cells. The plaques of rVSV-SARS-CoV, rVSV-SARS-CoV-2, and rVSV-G formed 5 days of post-infection on Vero cells. Above data are representative of three independent experiments. **e** Mutations acquired in the S protein by serial passaging at the 15th passage

SARS-CoV-2 and SARS-CoV are genetically close related coronaviruses using the same entry receptor ACE2. However, the pathology and immune responses developed by patients infected with SARS-CoV-2 and SARS-CoV are different. Here, we developed two replication-competent recombinant VSV viruses expressing the S of SARS-CoV-2 WH01 strain^35^ with a 21 amino acids (aa) deletion at the C-terminus (assigned as rVSV-SARS-CoV-2) and SARS-CoV BJ01 strain with a 19 aa at the C-terminus (assigned as rVSV-SARS-CoV)^12^ (Fig. 1a). The deletion of ~20 aa at the C-terminus of S protein is an established design to increase the titers of recombinant VSV-vectored coronaviruses.^22,23^ Two bands representing S and S1, 190 and 110 KDa, were detected from purified rVSV-SARS-CoV-2 and infected cell lysates. While for SARS-CoV, the S1 band was quite weak due to the lack of furin cleavage site (Fig. 1b). To assess the growth kinetics of rVSVs, viruses in the supernatant were measured every 12 h by a focus forming assay with anti-S antibodies. The peak titer of rVSV-SARS-CoV-2 and rVSV-SARS-CoV were 5.3 × 10^6^ FFU/ml (Focus-forming unit/ml) and 2 × 10^6^ FFU/ml, respectively (Fig. 1c). Plaques formed by rVSV-SARS-CoV-2 were smaller than those by rVSV-G. In contrast, no obvious plaques were formed by rVSV-SARS-CoV (Fig. 1d). Two mutations L455L (synonymous mutation) and A684D (outside of the RBD region) emerged in the S protein until passage 15 (Fig. 1e).

### Immunogenicity of rVSV-SARS-CoV-2 and rVSV-SARS-CoV

**Fig. 2:**
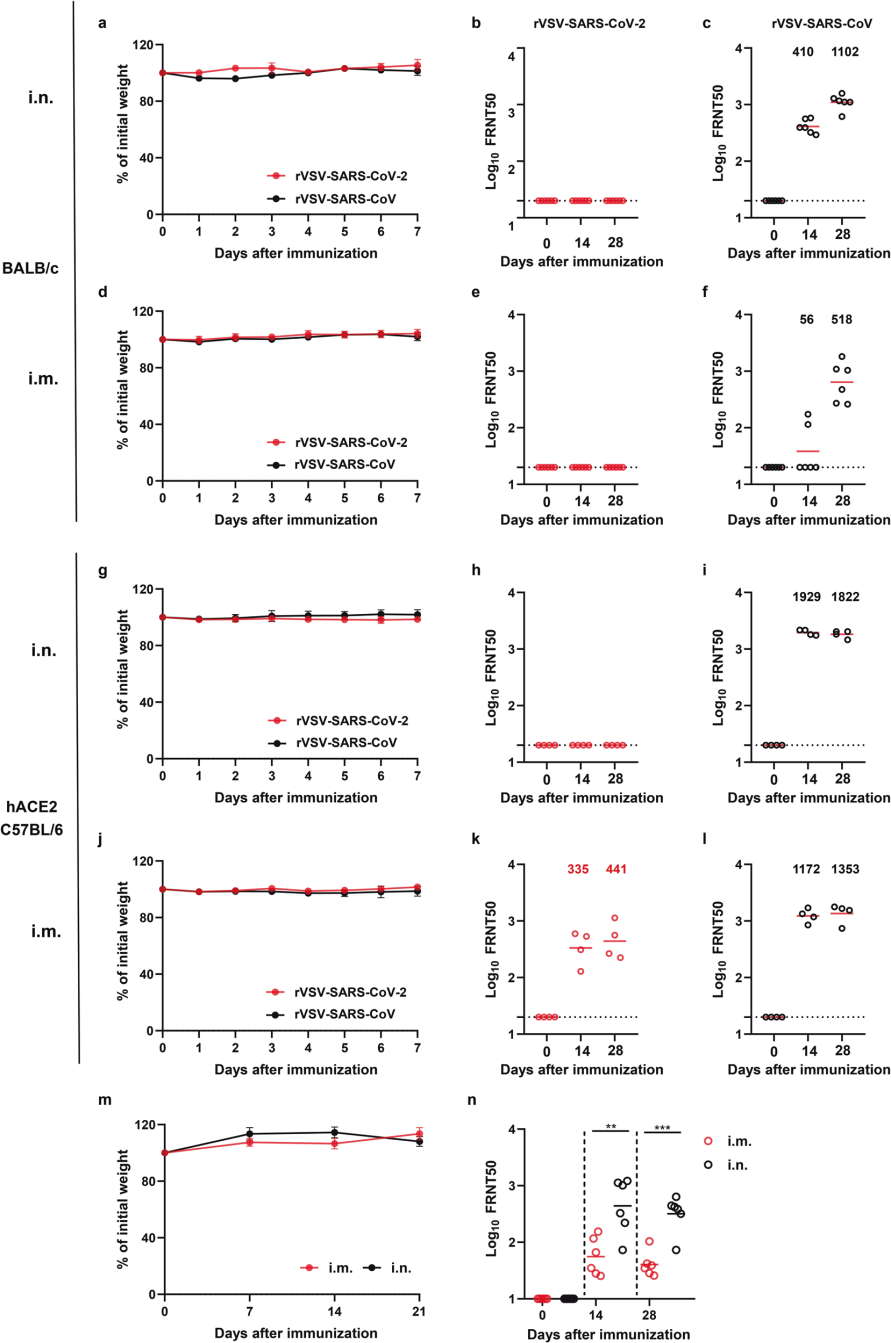
Immunogenicity of rVSV-SARS-CoV and rVSV-SARS-CoV-2. **a**–**f** Groups of female BALB/c mice (*n* = 6) or **g**–**l** hACE2 knock-in C57BL/6 mice (*n* = 4) were vaccinated with a single dose of rVSV-SARS-CoV-2 or rVSV-SARS-CoV via **a**–**c**, **g**–**i** i.n. (2 × 10^5^ FFU/animal) or **d**–**f**, **j**–**l** i.m. route (8 × 10^5^ FFU/animal). **a**, **d**, **g**, **j** Following vaccination, weight changes were monitored for 7 days. **b**, **c**, **e**, **f**, **h**, **i**, **k**, **l** NAb titers against rVSV-eGFP-SARS-CoV-2 (red circle) or rVSV-eGFP-SARS-CoV (black circle) were determined by FRNT and calculated by the Reed-Muench method at 14 days or 28 days of post-vaccination. Group geometric mean titers (GMTs) were indicated. **m**, **n** Groups of cynomolgus monkeys (*n* = 6) were immunized i.n. or i.m. with as single dose of rVSV-SARS-CoV-2 (5 × 10^6^ FFU/animal). **m** Body weight was monitored every week for three weeks. **n** The NAb titers were determined by FRNT at 14 and 28 days of post-immunization. Statistical significance was determined using unpaired two-tailed student’s *t*-test. **p < 0.01, ***p < 0.001. Error bars in **a**, **b**, **g**, **j**, **m** indicate standard deviation of the mean

Clinical reports suggest that SARS-CoV induces stronger humoral immune responses than SARS-CoV-2. Here we compared the immune responses induced by rVSV-SARS-CoV-2 and rVSV-SARS-CoV in animal models. We first immunized wild-type BALB/c mice with rVSV-SARS-CoV-2 or rVSV-SARS-CoV via the intranasal (i.n.) route at a dose of 2 × 10^5^ FFU or intramuscular (i.m.) route at a dose of 8 × 10^5^ FFU. Body weight was monitored for one week and no obvious loss was observed in all the groups (Fig. 2a, d). Robust neutralization activity was elicited by rVSV-SARS-CoV via both i.n. and i.m. routes. The geometric mean titers (GMTs) of serum NAbs of the i.n. group were 410 at 14 days and increased to 1102 at 28 days as measured by a FRNT assay (Fig. 2c). The titers in the i.m. group were about half of those in the i.n. group (Fig. 2f). On the other hand, no NAbs were detected at all in rVSV-SARS-CoV-2 immunized mice (Fig. 2b, e).

Murine ACE2 does not serve as an entry receptor for SARS-CoV-2 and SARS-CoV efficiently^36^ and wild-type mice are not susceptible to rVSV-SARS-CoV and rVSV-SARS-CoV-2. We next examined the immunogenicity of rVSV-SARS-CoV-2 and rVSV-SARS-CoV in hACE2 knock-in mice.^23^ Groups of hACE2 knock-in mice were vaccinated with rVSV-SARS-CoV-2 or rVSV-SARS-CoV using the same routes and dosage as the BALB/c mice. Body weight was monitored for 1 week and no obvious loss was observed in all groups (Fig. 2g, j). rVSV-SARS-CoV-2 elicited robust neutralizing activity against SARS-CoV-2 only via the i.m. route with titers of 335 at 14 days and 441 at 28 days. The sera from the i.n. group did not show specific neutralizing activity (Fig. 2h, k). In contrast, rVSV-SARS-CoV induced significantly high levels of NAbs against SARS-CoV via both vaccination routes (Fig. 2i, l). In the i.n. group, the NAb titers rapidly reached 1929 at 14 days and 1822 at 28 days. Slightly lower NAb responses were induced in the i.m. group with titers of 1172 at 14 days and 1353 at 28 days. Thus, rVSV-SARS-CoV was significantly more immunogenic than rVSV-SARS-CoV-2, especially via i.n. route. As for rVSV-SARS-CoV-2, only i.m. injection could elicit NAb responses.

### rVSV-SARS-CoV-2 is more effective by intranasal route in monkeys

We further tested the immune response of rVSV-SARS-CoV-2 in non-human primates. Groups of cynomolgus monkeys (*Macaca fascicularis*) (*n* = 6) were immunized via i.m. or i.n. route at a single dose of 5 × 10^6^ FFU rVSV-SARS-CoV-2. Body weight was monitored for 21 days and no obvious weight loss was observed (Fig. 2m). No rVSV-SARS-CoV-2 shedding was detected in blood, nasal secretion, urine and feces after immunization (Supplementary Fig. S1). Serum NAbs in the i.n. group were measured at 14 and 28 days, with titers of 440 and 320, respectively. However, the NAb titers in the i.m. group were only 56 and 40 at 14 and 28 days, respectively (Fig. 2n). Surprisingly, the NAb levels elicited via i.n. route were about 8-fold higher than those via i.m. injection, which was opposite to our results in hACE2 knock-in mouse models. Given that macaques are physiologically and phylogenetically closer to humans than the mice, we speculate that the i.n. route might be an optimal choice for human vaccination.

### The cross-reaction between rVSV-SARS-CoV-2 and rVSV-SARS-CoV

**Fig. 3:**
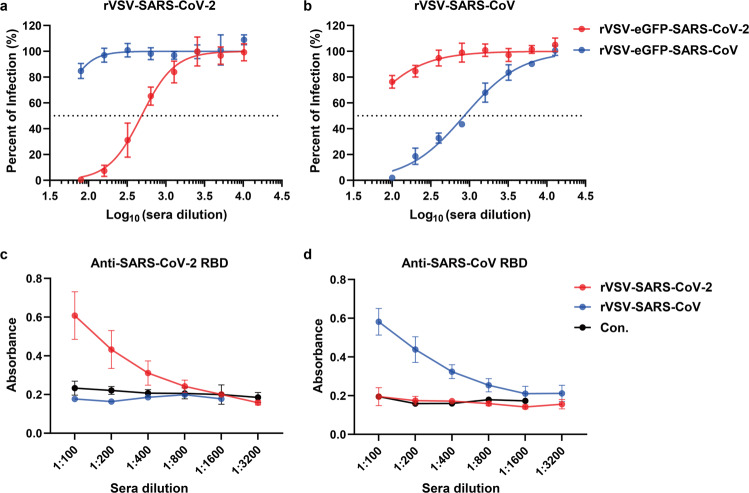
Antigenic cross-reaction between rVSV-SARS-CoV and rVSV-SARS-CoV-2. **a**, **b** Neutralization curves of sera from (Fig. 2k, l) against rVSV-eGFP-SARS-CoV-2 and rVSV-eGFP-SARS-CoV. **c**, **d** S RBD specific IgG in the sera from rVSV-SARS-CoV-2, rVSV-SARS-CoV or mock immunized hACE2 mice (Con.) was determined by ELISA with captured SARS-CoV-2 S RBD (**c**) or SARS-CoV S RBD (**d**). Error bars in **a**, **b**, **c**, **d** indicate standard deviation of the mean

The S proteins of SARS-CoV-2 and SARS-CoV share 76% amino acid identity. Thus, we would expect certain level of cross-reaction in the sera from immunized animals. However, the serum samples from mice vaccinated with rVSV-SARS-CoV or rVSV-SARS-CoV-2 did not show cross-neutralizing activities (Fig. 3a, b). We further investigated the antigenic cross-reaction of immunized sera with the captured RBD domains of S proteins using ELISA. Correspondingly, no cross-reactivity was detected either (Fig. 3c, d).

### Construction of vaccine candidate by expressing a chimeric antigen

**Fig. 4:**
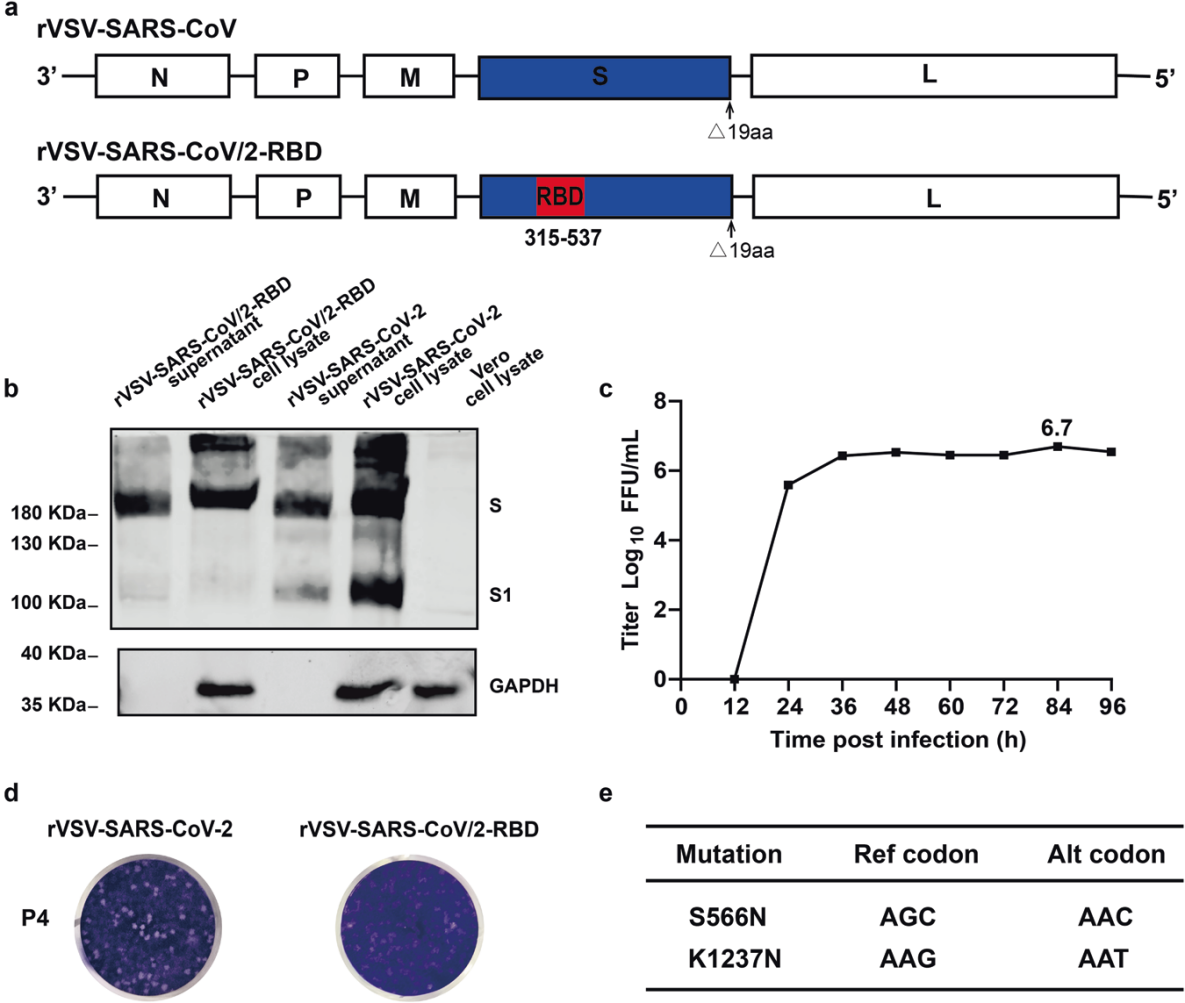
Characterization of the rVSV-SARS-CoV/2-RBD. **a** Schematic diagrams showing genome organization in rVSV vector. The SARS-CoV S RBD in rVSV-SARS-CoV was replaced by SARS-CoV-2 S RBD to generate rVSV-SARS-CoV/2-RBD. **b** Purified rVSV-SARS-CoV-2 or rVSV-SARS-CoV/2-RBD from supernatants (Lane 1 and 3), lysates of rVSV-SARS-CoV-2 and rVSV-SARS-CoV/2-RBD infected Vero cells, and mock infected Vero cells (Lane 2, 4 and 5) were blotted with an antibody recognizing the RBD domain of SARS-CoV-2 S protein, using GAPDH as input control. **c** Growth kinetics of rVSV-SARS-CoV/2-RBD. Vero cells were infected by the rVSV-SARS-CoV/2-RBD (MOI = 0.01) and virus titers in the supernatant were measured at the indicated time points post infection by immunofluorescence. **d** Plaque morphology of rVSV-SARS-CoV-2 or rVSV-SARS-CoV/2-RBD at 5 days of post-infection in Vero cells. **e** Mutations acquired in the S protein by serial passaging at the 15th passage

To enhance the immunogenicity of the vaccine candidate, we designed a chimeric antigen by replacing the S RBD domain of SARS-CoV-2 with the corresponding region in the S protein of SARS-CoV. The recombinant VSV expressing the chimeric antigen was rescued and named rVSV-SARS-CoV/2-RBD (Fig. 4a). In both purified viral particles and infected cell lysates, chimeric S protein was expressed as a single 190 KDa band as a result of lacking the furin cleavage site (Fig. 4b). The rVSV-SARS-CoV/2-RBD grew robustly in Vero cells with a peak titer of 5 × 10^6^ FFU/ml, similar as rVSV-SARS-CoV-2 (Figs. 1c and 4c). Plaques formed by rVSV-SARS-CoV/2-RBD were slightly smaller than those by rVSV-SARS-CoV-2 (Fig. 4d). To test the genetic stability, rVSV-SARS-CoV/2-RBD was serially passaged in Vero cells and the whole viral genome was sequenced every five passages. Two mutations S566N and K1237N (outside of the RBD region) emerged in S protein until passage 15 (Fig. 4e).

### Efficacy of the rVSV-SARS-CoV/2-RBD in rodent and non-human primate models

**Fig. 5:**
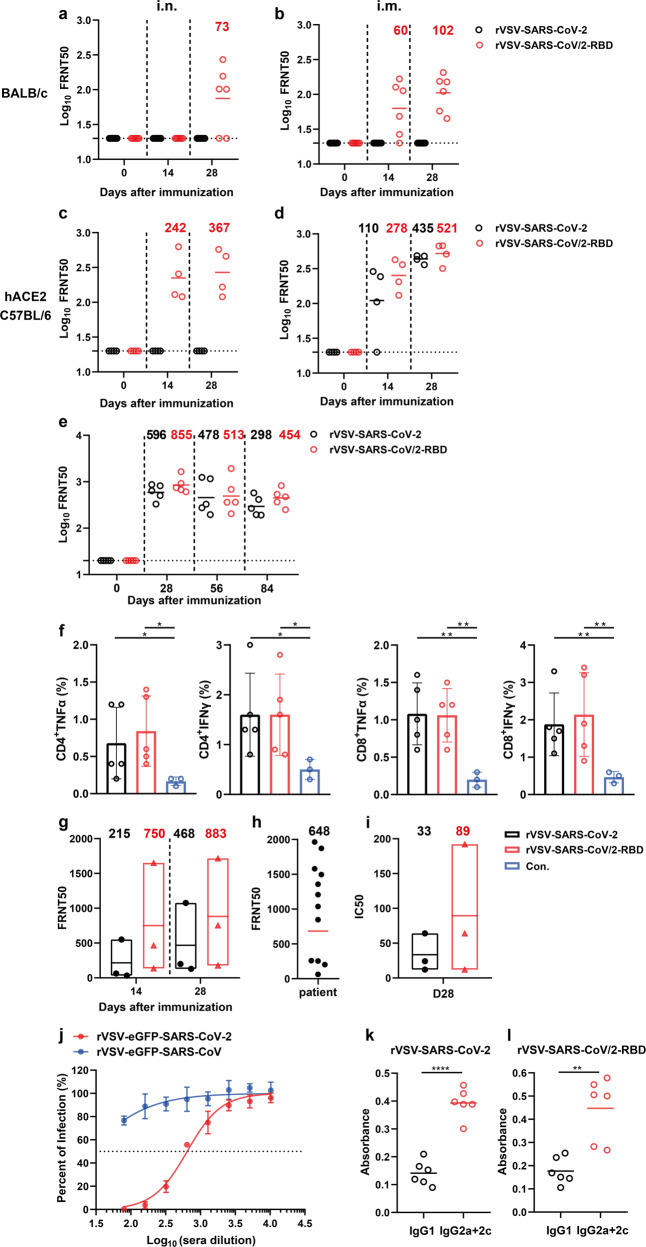
Immunogenicity of rVSV-SARS-CoV/2-RBD in mice and Chinese rhesus macaques. **a**, **b** Groups of female BALB/c mice (*n* = 6) or **c**, **d** female hACE2 knock-in C57BL/6 mice (*n* = 4) were immunized with a single dose of rVSV-SARS-CoV-2 (black) or rVSV-SARS-CoV/2-RBD (red) via i.n. (10^5^ FFU/animal) (**a**, **c**) or i.m. route (4 × 10^5^ FFU/animal) (**b**, **d**). **a**–**d** NAb titers against rVSV-eGFP-SARS-CoV-2 were determined by FRNT and calculated by the Reed-Muench method at 14 or 28 days of post immunization. GMTs were indicated. **e** Groups of female hACE2 knock-in C57BL/6 mice (*n* = 5) were immunized with a single dose of rVSV-SARS-CoV-2 (black) or rVSV-SARS-CoV/2-RBD (red) via i.m. route (8 × 10^5^ FFU/animal). NAb titers against rVSV-eGFP-SARS-CoV-2 were determined by FRNT and calculated by the Reed-Muench method at day 28, 56, or 84 of post-immunization. GMTs were indicated. **f** Percentage of TNFα and IFNγ expressing CD4 and CD8 cells after SARS-CoV-2 RBD peptide stimulation. Statistical significance was determined using unpaired two-tailed student’s *t*-test. **p < 0.01, *p < 0.05 (**g**, **i**). Groups of Chinese rhesus macaques (*n* = 3) were i.n. immunized with rVSV-SARS-CoV-2 or rVSV-SARS-CoV/2-RBD (5 × 10^6^ FFU/animal). NAb titers were determined by **g** FRNT and **i** live SARS-CoV-2 neutralization assay. **h** The titers of NAb in sera from twelve COVID-19 convalescent patients against rVSV-eGFP-SARS-CoV-2 were determined by FRNT. **j** Neutralization curves of sera from **d** against rVSV-eGFP-SARS-CoV-2 and rVSV-eGFP-SARS-CoV. **k**, **l** Indicated IgG subclass (IgG1, IgG2a, and IgG2c) of SARS-CoV-2 S RBD specific antibodies were determined by ELISA. Statistical significance was determined using one-way ANOVA Kruskal–Wallis test. ****p < 0.0001, **p < 0.01. Error bars in **f**, **j** indicate standard deviation of the mean

To test the immunogenicity of chimeric antigen, we first immunized wild-type BALB/c mice with rVSV-SARS-CoV-2 or rVSV-SARS-CoV/2-RBD intranasally (10^5^ FFU) or intramuscularly (4 × 10^5^ FFU) per animal. Body weight was monitored for 7 days after vaccination and no weight loss was observed (Supplementary Fig. S2a, b). Compared to rVSV-SARS-CoV-2, which failed to elicit NAbs in wild-type BALB/c mice, rVSV-SARS-CoV/2-RBD induced detectable serum neutralizing activities via both i.n. and i.m. routes (Fig. 5a, b).

We then determined the immunogenicity of rVSV-SARS-CoV/2-RBD in the hACE2 knock-in mouse model with a same immunizing protocol. No weight loss was observed within 7 days after immunization (Supplementary Fig. S2c, d). Via i.m. route, the NAb titers elicited by the chimeric rVSV-SARS-CoV/2-RBD were 4.0-fold and 1.9-fold higher than those by rVSV-SARS-CoV-2 at 14 and 28 days, respectively (Fig. 5c, d). The rVSV-SARS-CoV/2-RBD also induced a strong NAb response via the i.n. route, exhibiting enhanced immunogenicity compared to rVSV-SARS-CoV-2 (Fig. 5c, d). The NAb titers in the sera were monitored for three months to test the durability and a similar decrease of less than two-fold was observed in both rVSV-SARS-CoV-2 and rVSV-SARS-CoV/2-RBD groups (Fig. 5e).

To determine whether the vaccine candidate elicited cellular immune response, hACE2 knock-in mice were immunized via i.m. route with one dose of 8 × 10^5^ FFU rVSV-SARS-CoV-2 or rVSV-SARS-CoV/2-RBD respectively. Splenocytes were harvested on day 7 and stimulated ex vivo with a pool of 53 overlapping 15-mer S-RBD peptides. Significantly enhanced TNFα and IFNγ levels were detected in both rVSV-SARS-CoV-2 or rVSV-SARS-CoV/2-RBD groups as compared with mock control (Fig. 5f).

We further evaluated the immunogenicity of rVSV-SARS-CoV/2-RBD in Chinese rhesus macaques. Three 20-year-old animals per group were i.n. immunized with 5 × 10^6^ FFU rVSV-SARS-CoV-2 or rVSV-SARS-CoV/2-RBD. NAb titers elicited by rVSV-SARS-CoV/2-RBD were 750 and 883 at 14 and 28 days, which were 3.5-fold and 1.9-fold higher than those by rVSV-SARS-CoV-2, respectively (Fig. 5g). The level of NAbs induced in convalescent sera from twelve COVID-19 patients determined by the same FRNT assay (GMT: 648) was lower than that induced by rVSV-SARS-CoV/2-RBD in macaques (Fig. 5h). The serum neutralizing activities at 28 days were also determined using a live virus neutralization assay (Fig. 5i). NAb titers in the rVSV-SARS-CoV/2-RBD vaccinated group were 89, which were 2.7-fold higher than those in the rVSV-SARS-CoV-2 group and higher than the average titer of 50 in convalescent COVID-19 patients.^37^ These results confirmed that rVSV-SARS-CoV/2-RBD is more immunogenic than rVSV-SARS-CoV-2 in both mouse and macaque models.

To determine whether the rVSV-SARS-CoV/2-RBD could elicit NAbs against SARS-CoV, sera from rVSV-SARS-CoV/2-RBD immunized hACE2 mice were tested against rVSV-eGFP-SARS-CoV-2 and rVSV-eGFP-SARS-CoV. As shown in Fig. 5j, the sera did not cross-neutralize rVSV-eGFP-SARS-CoV.

Golden Syrian hamster (*Mesocricetus auratus*) is a rodent model susceptible to SARS-CoV-2 and have been used in the evaluation of rVSV based COVID-19 vaccines.^22^ The immunogenicity of the two vaccine candidates was further determined in the hamsters immunized with two doses of 10^5^ FFU rVSV-SARS-CoV/2-RBD or rVSV-SARS-CoV-2 per animal (Supplementary Fig. S3a). The NAb levels and RBD-specific IgG were measured at 28 days of post-immunization. The rVSV-SARS-CoV/2-RBD induced a strong humoral immune response via the i.n. route, exhibiting enhanced immunogenicity compared to rVSV-SARS-CoV-2 (Supplementary Fig. S3b, c).

### Antibody isotype profiles elicited by rVSV-SARS-CoV-2 and rVSV-SARS-CoV/2-RBD

T helper 2 cells (Th2) biased immune responses correlate with vaccine-associated enhanced respiratory disease.^38^ The Th1/Th2 balance was thus evaluated after immunization by comparing the level of RBD-specific immunoglobulins, IgG2a and IgG2c, and IgG1, which are surrogates of Th1 and Th2 responses, respectively. Significantly higher levels of IgG2a plus IgG2c were produced by rVSV-SARS-CoV-2 (Fig. 5k) and rVSV-SARS-CoV2-RBD (Fig. 5l) in all the hACE2 knock-in mice, as opposed to low levels of IgG1, indicating a safe Th1 biased immune response.

### rVSV vaccines protect hACE2 mice against SARS-CoV-2

**Fig. 6:**
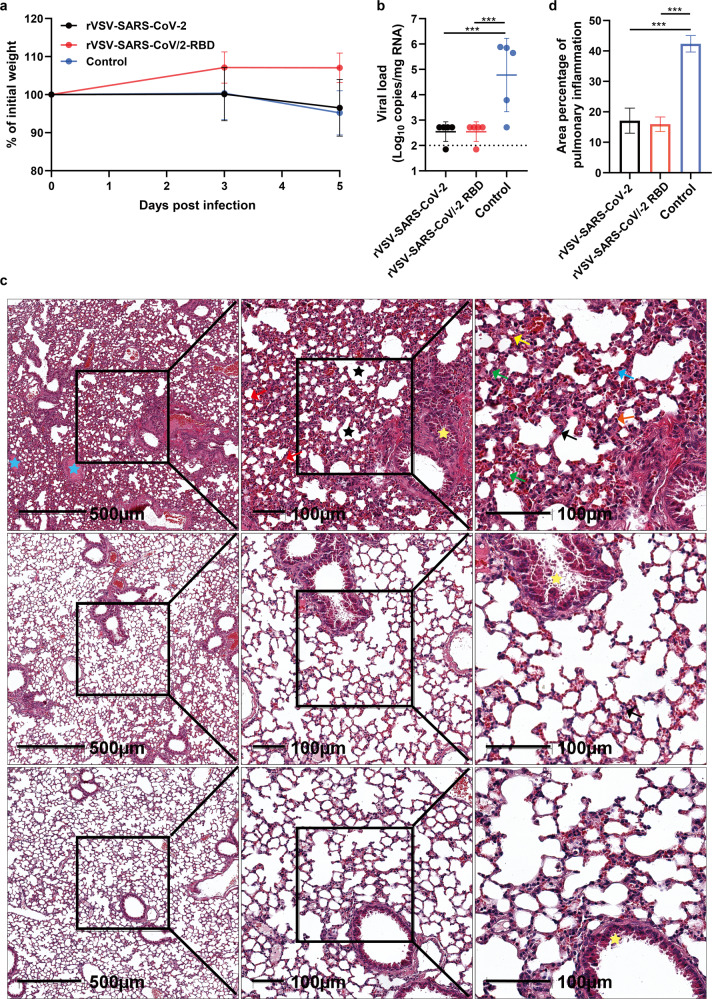
Protection efficacy of rVSV-SARS-CoV-2 or rVSV-SARS-CoV/2-RBD in hACE2 mice following SARS-CoV-2 challenge. **a**–**d** At 50 days after immunization with a single i.m. dose of rVSV-SARS-CoV-2, rVSV-SARS-CoV/2-RBD (4 × 10^5^ FFU/animal) or DMEM (Con.), hACE2 knock-in mice (*n* = 5) were i.n. challenged with 5 × 10^5^ TCID50 of SARS-CoV-2. **a** Body weight was monitored for 5 days of post-challenge. **b** Viral loads in lung tissue were determined by qRT-PCR at 5 days after SARS-CoV-2 infection. Statistical significance was determined using two-way ANOVA with multiple comparisons. ***p < 0.001. Dot line indicates detection limit. **c** Representative hematoxylin and eosin (H&E) staining of lung sections from SARS-CoV-2 infected mice at 5 days of post-infection. **d** The area percentage of pulmonary inflammation was estimated by pathological quantitative analysis using Halo v3.2 Classifier DenseNet AI (Plugin) in three groups. Error bars in **a**, **b**, **d** indicate standard deviation of the mean

Humoral immune response elicited by rVSV-SARS-CoV/2-RBD and rVSV-SARS-CoV-2 via i.m. route was significantly higher than that via i.n. route in mouse models. To assess whether our vaccine candidates can confer protection against SARS-CoV-2, groups of hACE2 knock-in mice (*n* = 5) were i.n. challenged with 5 × 10^5^ TCID50 SARS-CoV-2 at 50 days after a single i.m. shot of 4 × 10^5^ FFU rVSV-SARS-CoV/2-RBD or rVSV-SARS-CoV-2. Body weight was monitored at 3 and 5 days after challenge. The group received rVSV-SARS-CoV/2-RBD showed a slight weight increase of 7%, while the rVSV-SARS-CoV-2 and control groups displayed similar weight loss (3.4 and 4.8%) at 5 days after challenge (Fig. 6a). The mice were sacrificed for viral load test and pathogenesis analysis at 5 days. One lung of each mouse was homogenized to analyze viral load by real-time PCR. The lung viral RNA load was high in four out of five control animals. Viral RNA levels of the groups received rVSV-SARS-CoV/2-RBD or rVSV-SARS-CoV-2 were 2.2 logs lower than the control group, which were close to the limit of detection (Fig. 6b).

Lung sections were stained with hematoxylin and eosin (H&E) (Fig. 6c). Lung sections from the control group showed interstitial pneumonia with thickened alveolar septa and infiltration of inflammatory cells. HE staining showed there was a degree of consolidation in the lung tissues, characterized by diffuse alveolar damage with a small amount of serous and fibrin in the alveolar cavities (green arrow). The lungs also displayed type II alveolar epithelial cell proliferation (blue arrow), alveolar septa thickening (black arrow), alveolar interstitial capillary congestion (red arrow), and infiltrating inflammatory cells-including macrophages (yellow arrow), lymphocytes (pink arrow) and neutrophils (orange arrow). Partial alveolar septa rupture, cystic cavity formation (black star), peri-vascular inflammation and intravascular coagulation (blue star), mucosal epithelium exfoliation of bronchioles and exudate in the lumen of the bronchiole (yellow star) were observed. In lung sections from animals that received rVSV-SARS-CoV-2, the alveolar capillaries were slightly congested, with a small amount of red blood cells (black arrow) in the alveolar cavities. Some epithelial cells of bronchiole were lightly sloughed (yellow star). In the group immunized with rVSV-SARS-CoV/2-RBD, the lungs were normal by morphological observation, except some epithelial cells of the bronchioles were mildly desquamated (yellow star) (Fig. 6c). The area percentage of pulmonary inflammation was further quantitatively analyzed using Halo v3.2 Classifier DenseNet AI (Fig. 6d). The percentage of the pulmonary inflammatory area was 42.35, 17.10, and 15.93% in the control group, rVSV-SARS-Cov-2 group, and rVSV-SARS-Cov/2-RBD group, respectively. These results suggest that the two VSV-based vaccines candidates generated protective immunity, which limited SARS-CoV-2-caused lung disease in mice.

## Discussion

In the past 20 years, three coronaviruses, SARS-CoV, MERS-CoV, and SARS-CoV-2, emerged and threatened human health and public safety. Universal and efficacious vaccines are in urgent need for the control of emerging and future coronaviruses.^39^ By comparing the immune responses induced by rVSV-SARS-CoV-2 and rVSV-SARS-CoV, we found the cross-neutralization between SARS-CoV and SARS-CoV-2 is very limited, indicating the difficulty in the development of a universal coronavirus vaccine. We also found i.n. vaccination of rVSV-SARS-CoV-2 elicited significantly improved NAbs responses than i.m. route in macaques, providing the basis for developing nasal-spray rVSV-SARS-CoV-2 vaccine. Because rVSV-SARS-CoV induced much higher humoral immune responses than rVSV-SARS-CoV-2, we developed a chimeric SARS-CoV-2 vaccine using the SARS-CoV S protein as backbone to express the S RBD of SARS-CoV-2. The resulting chimeric vaccine not only elicited high level of NAbs, but conferred protection in mouse model.

Since the initial outbreak in late 2019, COVID-19 vaccine development has proceeded at record speed. As of June 2021, several COVID-19 vaccines such as mRNA vaccine, inactivated vaccine, and adenovirus-based vaccine have been approved for emergency use in some countries. Two VSV vector-based vaccine candidates were in clinical trials, both of which express the full-length S protein of SARS-CoV-2 with the C-terminal 21 aa deleted. On January 25, 2021, Merck ended the clinical trial of a VSV-based COVID-19 vaccine. In this trial (ClinicalTrials.gov Identifier: NCT04569786), a single dose of vaccine was administered via i.m. injection with dosage levels ranging from 5 × 10^5^ to 5.55 × 10^7^ PFU. However, the vaccine induced immune responses were inferior to natural infection and other COVID-19 vaccines. Yahalom-Ronen et al. from the Israel Institute for Biological Research also developed a VSV-based vaccine using the same strategy, which is currently in phase 2 clinical trial.^22^ In this trial, i.m. immunization was also applied yet the results have not been released. Live vector-based vaccines must infect a certain number of cells to induce immune responses. The entry of rVSV-SARS-CoV-2 and rVSV-SARS-CoV is mediated by the heterogenous S proteins engaging the major cellular receptor ACE2.^35^ Immunohistochemical analysis revealed that there are significant higher levels of ACE2 in the respiratory tract than in skeletal muscle tissues.^40^ Furthermore, the nasal tissue is highly susceptible to SARS-CoV-2,^41^ suggesting that the i.n. route might be more suitable for administration of VSV-based COVID-19 vaccine than the i.m. route. In our study, i.n. vaccination of macaques with 5 × 10^6^ FFU generated 8-fold higher titers of NAbs than i.m. injection, highlighting the importance of immunization route. Additionally, intranasal administration makes self-vaccination possible during emergency situations, thus reducing the burden of medical suppliers and speeding the vaccination process.

SARS-CoV-2 belongs to a diverse virus family including thousands of viruses infecting a big range of domestic and wild animals. SARS-CoV emerged in 2002 with a fatality of 10%; MERS-CoV emerged in 2012 with 34% fatality. The potential for new coronaviruses to emerge and cause pandemics is high. Thus, we need a universal coronavirus vaccine to cope with the next coronavirus.^39^ However, this is not easy. SARS-CoV and SARS-CoV-2 share 76% amino acid identity in the S protein and certain levels of cross-reactivity between SARS-CoV and SARS-CoV-2 patient serum has been reported.^42,43^ Unfortunately, we did not observe cross-reaction or cross-neutralization between serum samples from rVSV-SARS-CoV and rVSV-SARS-CoV-2 immunized animals. RBD is the major antigen in the S protein and elicits NAbs that block the interaction with ACE2 receptor. Structural studies have identified key residues in the S protein that interact with ACE2, including Leu455, Phe456, Phe486, Gln493, Gln498, and Asn501 for SARS-CoV-2 and Tyr442, Leu443, Leu472, Asn479, Tyr484, and Thr487 for SARS-CoV.^15^ However, only Gln498 of SARS-CoV-2 and Tyr484 of SARS-CoV interact with the same epitope in ACE2 including Asp38, Tyr41, Gln42, Leu45, and Lys353. This might explain the low cross-reaction between SARS-CoV and SARS-CoV-2. The N-terminal domain (NTD) of the S protein is another antigen that can elicit neutralizing antibodies against SARS-CoV-2. Some high potent NAbs were identified from convalescent COVID-19 patients. However, NTDs of SARS-CoV-2 and SARS-CoV only share 62.6% consensus positions and 49.3% identity positions. And, NTD-directed NAbs predominantly recognize a single supersite, While RBD-directed NAbs recognize non-overlapping epitopes.^44,45^ Thus, NTD-directed antibodies are unlikely to contribute to the cross-reactivity between SARS-CoV-2 and SARS-CoV.

Clinical surveillances suggest serum neutralizing activities in the convalescent SARS patients of SARS-CoV are stronger than those in SARS-CoV-2 and last for longer time.^25–31^ This was consistent with our data that rVSV-SARS-CoV could elicit stronger humoral immune response than rVSV-SARS-CoV-2 in mice. Given that the S RBD domain is the major immunogen for coronavirus and the S protein of SARS-CoV is more immunogenic, we designed a chimeric rVSV- SARS-CoV/2-RBD to increase the immunogenicity. As predicted, the chimeric vaccine stimulated more rapid and efficient NAb responses than rVSV-SARS-CoV-2 in both the BALB/c and hACE2 knock-in mice. In contrast, rVSV-SARS-CoV-2 failed to produce NAbs in BALB/c mice regardless of the immunization routes we studied. In hACE2 knock-in mice, only i.m. injection induced NAbs. In the rhesus macaques, a single i.n. vaccination of rVSV-SARS-CoV/2-RBD produced NAbs with a mean titer of 883 at 28 days, which was ~2-fold higher than rVSV-SARS-CoV-2 and higher than the NAb titers in the convalescent COVID-19 patients.

Antibody dependent enhancement (ADE) of SARS-CoV-2 has been hypothesized at the beginning of the COVID-19 pandemic. To date, after hundreds of millions of natural infections and human immunizations in the real world, there is no in vitro and in vivo evidence supporting this hypothesis.^46^ However, Liu et al. reported the potential ADE caused by MVA-vectored vaccine against SARS-CoV in Chinese macaques.^47^ Here, our results suggest that there is no cross-neutralizing activity against SARS-CoV in serum raised by rVSV-SARS-CoV/2-RBD. Further, high levels of IgG2a and IgG2c were observed in the mice serum immunized by rVSV-SARS-CoV/2-RBD through antibody isotype profile analysis, indicating the induction of a safe Th1-biased immune response with low risk of ADE.

VSV-based COVID-19 vaccine candidates may offer protection against SARS-CoV-2 in hACE2 transgenic mice and hamster model systems via i.m. injection.^22,23^ In this study, we demonstrated that a single i.m. dose of rVSV-SARS-CoV-2 or rVSV-SARS-CoV/2-RBD protected the hACE2 knock-in mice against SARS-CoV-2 challenge with very mild pathogenesis and two log reduction of viral loads in the lung. Animals received rVSV-SARS-CoV/2-RBD showed a 7.0% weight increase after SARS-CoV-2 challenge, while those immunized by rVSV-SARS-CoV-2 with 3.4% weight loss, suggesting the better protective efficacy of rVSV-SARS-CoV/2-RBD. These results pave the road for further application of rVSV-SARS-CoV/2-RBD as an efficacious vaccine candidate for SARS-CoV-2. The strategy of chimeric expression SARS-CoV-2 S RBD in SARS-CoV represents a new design for other vaccine platforms.

## Materials and methods

### Ethics statement

The rVSV studies were conducted under biosafety level 2 (BSL2) conditions. Research with live SARS-CoV-2 was performed in a biosafety level 3 (BSL3) facility. All animal studies were carried out in strict accordance with the recommendations in the Guide for the Care and Use of Laboratory Animals of the Ministry of Science and Technology of the People’s Republic of China. The protocols for animal studies were approved by the Committee on the Ethics of Animal Experiments of the Institute of Zoology, Chinese Academy of Sciences (Approval number: IOZ-IACUC-2020–036). Virus inoculations were performed under anesthesia that was induced and maintained with Isoflurane, and all efforts were made to minimize animal suffering.

### Cells and antibodies

Vero cells (African green monkey kidney cells) and HEK293T (human embryonic kidney cells) were obtained from American Type Culture (ATCC) and maintained in Dulbecco’s modified Eagle’s medium (DMEM) supplemented with 8% fetal bovine serum (FBS), 1% l-glutamine, and 1% penicillin-streptomycin. All cells were incubated at 37 °C with 5% CO_2_. Rabbit anti-SARS-CoV-2 and SARS-CoV S RBD polyclonal antibody was purchased from Sino Biological Inc.

### Construction, rescue, and characterization of rVSV viruses

The rVSV vector was designed, synthesized, and constructed as described previously.^35^ The humanized S protein coding sequence of SARS-CoV-2 Wuhan-Hu-1 strain (GenBank: YP_009724390.1) or SARS-CoV BJ01 strain (GenBank: AY278488.2) were synthesized by Genewiz Suzhou and inserted between the MluI and NotI sites into rVSV-ΔG plasmid. The resulting plasmids were named pVSV-SARS-CoV-2 or pVSV-SARS-CoV with VSV glycoprotein G coding sequence being replaced by that of SARS-CoV-2 or SARS-CoV S protein. The S RBD coding sequence of pVSV-SARS-CoV (315–537 aa) was replaced by that of the S RBD of SARS-CoV-2 (319–541 aa) and the resulting plasmid was named pVSV-SARS-CoV/2-RBD. S protein coding sequence in pVSV-eGFP-SARS-CoV-2 was replaced by that of SARS-CoV BJ01 strain lacking C-terminal 19 aa to generate pVSV-eGFP-SARS-CoV.

The rVSVs were rescued by a reverse genetics approach. Briefly, HEK293T cells were transfected with pVSV plasmids and supporting plasmids encoding T7 polymerase and N, P, M, G, L of VSV using a calcium phosphate method.^48^ rVSVs in the supernatants were collected and stored at −80 °C.

### Growth kinetics of rVSVs

Vero cells (4 × 10^6^) in T75 flasks were inoculated with rVSVs (MOI = 0.01). After 3 h, the culture medium was replaced with DMEM plus 2% FBS. The cells were grown at 28 °C, and the supernatant was harvested every 12 h. Titration was performed using a focus-forming assay on Vero cells.

### Animal experiments

The hACE2 knock-in mouse model was developed by the Institute of Zoology, Chinese Academy of Sciences, Beijing, China. Briefly, the donor vector containing homology arm sequence and hACE2 cDNA with BGH PolyA sequence, the specific sgRNA and cas9 mRNA were mixed and microinjected into the pronuclei of C57BL/6 zygotes, then the microinjected zygotes were transferred into oviducts of pseudopregnant foster mother mice. The hACE2 cDNA was inserted into the first coding sequence of mouse ACE2 (mACE2), so its expression was under the control of mouse ACE2 promoter.

Six to eight weeks-old female BALB/c mice, hACE2 knock-in C57BL/6 mice or golden Syrian hamsters (*Mesocricetus auratus*) were immunized with a single dose of rVSVs viruses via i.n. (50 μl per mouse) or i.m. (200 μl per mouse) route. Morbidity and weight were monitored for 7 d post vaccination. Serum samples were collected 14, 28, 56 or 84 d post vaccination for evaluating NAb titers against rVSV-eGFP-SARS-CoV-2 or rVSV-eGFP-SARS-CoV. Fifty days post vaccination, hACE2 knock-in C57BL/6 mice were challenged with 5 × 10^5^ TCID50 of SARS-CoV-2, and monitored for 5 d. Five days post challenge, mice were sacrificed and lung sample were collected for virus detection and pathology evaluation.

Groups of 2-to-6-years-old cynomolgus monkeys (*Macaca fascicularis*) and 20-years-old Chinese rhesus macaques (*Macaca mulatta*) were used in this study. Macaques were i.m. or i.n. immunized with indicated rVSVs by a single dose of 5 × 10^6^ FFU and monitored for morbidity and weight loss. Sera were collected 14 and 28 d post vaccination for evaluation of NAb titers against rVSV-eGFP-SARS-CoV-2 or live SARS-CoV-2.

### Neutralization assay

SARS-CoV-2 neutralization test was performed in a certified BSL3 laboratory. Vero cells were seeded in 96-well plates and reached ~90% confluence at the time of infection. Sera were heat-inactivated at 56 °C for 30 min. Two-fold serial dilutions of sera were mixed with equal volumes of viral solution to a final concentration of 100 TCID_50_/well and incubated at 37 °C for 1 h. The virus-serum mixtures (100 µl/well) were loaded on 96-well plates and incubated at 37 °C. The cytopathic effect (CPE) of each well was evaluated under a microscope and the NAb titer was recorded as the highest dilution of serum that showed 50% inhibition activity of SARS-CoV-2 (IC50).

For the focus reduction neutralization test (FRNT), 100–1000 FFU of rVSVs were incubated with five-fold serially diluted, heat-inactivated sera at room temperature (RT) for 30 min. The mixtures were then layered onto cells in 96-well plates. After 3 h, culture media were removed, and fresh DMEM containing 2% FBS and 20 mM NH_4_Cl was added. GFP positive cells were counted 20 h after infection using a fluorescent microscope or Opera Phenix High Content Screening System (PerkinElmer, Waltham, MA, USA). Neutralization titers were calculated as 50% inhibition of virus infection (FRNT50) using the Reed-Muench method.

### Peptide restimulation and intracellular cytokine staining

Splenocytes from intramuscularly vaccinated mice were incubated in culture with a pool of 53 overlapping 15-mer SARS-CoV-2 RBD peptides (jpi: PM-WCPV-S-RBD) for 6 h at 37 °C. Following blocking with 0.1% BSA-PBS, cells were stained on ice with CD4 APC/Cy7 (Biolegend: 100413), CD8 PerCP (Biolegend: 100713), and Zombie NIR Fixable Viability Kit (Biolegend: 423105). Stained cells were fixed and permeabilized with the Cyto-Fast™ Fix/Perm Buffer Set (Biolegend: 426803). Subsequently, intracellular staining was performed with anti-IFN-γ Brilliant Violet 421 (Biolegend: 505829) and anti-TNF-α PE (Biolegend: 506305). Analysis was performed on a BD FACSCalibur, using FlowJo X10.0 software.

### Enzyme-linked immunosorbent assay (ELISA)

ELISA plates were coated with 100 ng per well of SARS-CoV S RBD or SARS-CoV-2 S RBD protein (Sino Biological Inc., China) in coating buffer (50 mM sodium carbonate/bicarbonate, pH 9.6) at 4 °C for 12 h. After standard washing and blocking, plates were incubated with serial dilutions of heat-inactivated sera for 1 h at 37 °C. Following washes, the plates were incubated with anti-mouse IgG, IgG1, or IgG2a and IgG2c-horserdish peroxidase conjugates for 1 h at RT. After three washes, 3,5,3′5′-tetramethylbenzidine (TMB) (TIANGEN BIOTECH (BEIJING) CO., LTD) was added to each well and incubated at RT until the desired color density was reached. The reaction was stopped by adding 2 M sulfuric acid and the absorbance was measured at 450 nm using a SpectraMax i3 multi-mode microplate reader (Molecular Devices, San Jose, CA, USA).

### Histopathology

For hematoxylin and eosin (H&E) general histopathology evaluation, lungs were rapidly isolated, and fixed in 4% neutral-buffered PFA at RT for 7 days followed by routine processing for paraffin embedding. Coronal, serial sections, 4–5 µm thick, were performed and selected sections were stained with H&E for light microscopy examination. Images were acquired using a Nikon Eclipse 50i Light Microscope (Nikon, Tokyo, Japan) or Olympus BX60 microscope (Shinjuku, Tokyo, Japan).

### Tissues viral load determination

Animal tissues were weighed and homogenized at 5 days of post-infection. Tissue homogenates were clarified by centrifugation at 10,000 rpm for 5 min and stored at −80 °C. RNA was extracted using TRIzol Reagent (Thermo Fisher Scientific, USA) according to the manufacturer’s protocol. Quantification viral genomic copies was performed using One Step TB Green™ PrimeScript™ RT-PCR Kit (TaKaRa, Japan) on Applied Biosystems QuantStudio (Thermo Fisher Scientific, USA). Each sample was measured in triplicate. Primers used for qRT-PCR were: 5′-TCGTTTCGGAAGAGACAGGT-3′ (forward primer) and 5′-GCGCAGTAAGGATGGCTAGT-3′ (reverse primer). Viral loads were calculated as the viral genomic copies in one gram tissue.

## Supplementary information

Supplementary Materials for Enhanced protective immunity against SARS-CoV-2 elicited by a VSV vector expressing a chimeric spike protein

## Data Availability

The data supporting the findings of this study are included in this paper and its supplementary information.
