# Supplementary material for: Enhanced protective immunity against SARS-CoV-2 elicited by a VSV vector expressing a chimeric spike protein
Source: Signal Transduct Target Ther. 2021 Nov 10;6:389. doi: 10.1038/s41392-021-00797-9 (PMC8578532; doi:10.1038/s41392-021-00797-9)
Supplement: Supplementary file 1 — Supplementary Materials for Enhanced protective immunity against SARS-CoV-2 elicited by a VSV vector expressing a chimeric spike protein [file 41392_2021_797_MOESM1_ESM.docx]

Supplementary Materials for

Enhanced protective immunity against SARS-CoV-2 elicited by a VSV vector expressing a chimeric spike protein

Hongyue Li^†1,2^, Yuhang Zhang^†1,2^, Dong Li^†3^, Yong-Qiang Deng^†4^, Hongde Xu^†5^, Chaoyue Zhao^1,2^, Jiandong Liu^3^, Dan Wen^1,2^, Jianguo Zhao^6^, Yongchun Li^5^, Yong Wu^7^, Shujun Liu^8^, Jiankai Liu^3^, Junfeng Hao^8^, Fei Yuan^1^, Shuguang Duo*^9^, Cheng-Feng Qin*^4^, Aihua Zheng*^1,2,10^

Correspondence to: [zhengaihua@ioz.ac.cn](mailto:zhengaihua@ioz.ac.cn)

**This PDF file includes:**

Figures. S1 to S3

**
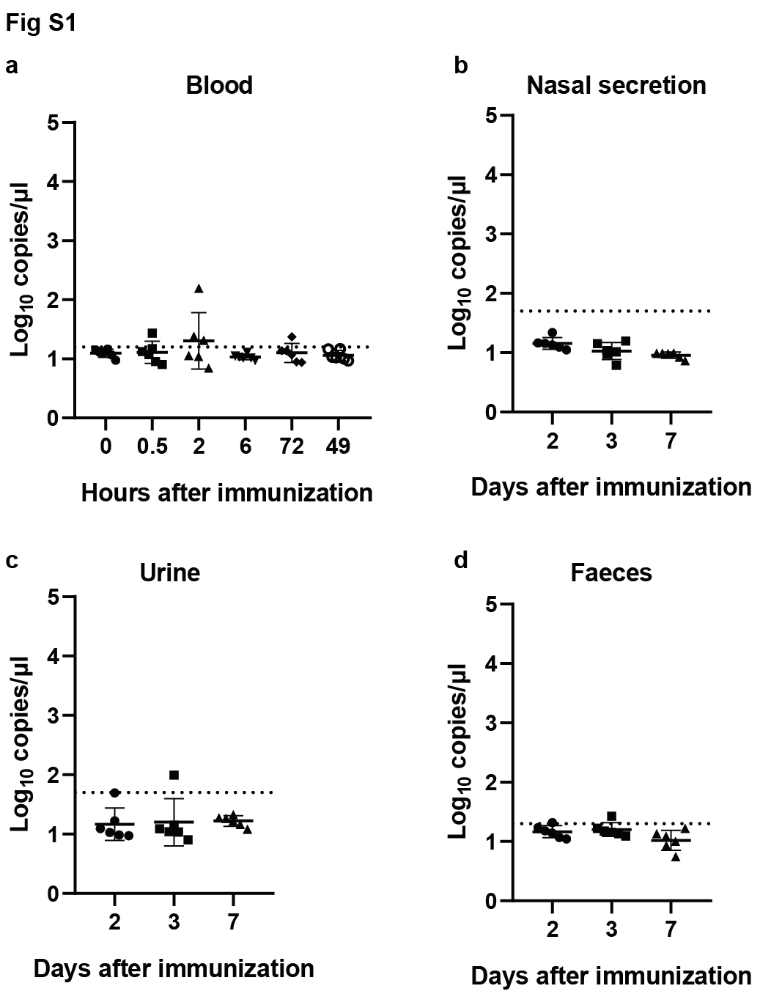
**

**Figure. S1. Viral shedding in monkeys during rVSV-SARS-CoV-2 vaccination. (a–d)** Groups of cynomolgus monkeys (n = 6) were immunized i.m. with as single dose of rVSV-SARS-CoV-2 (5 x 10^6^ FFU/animal). Viral loads in blood (a), nasal secretion (b), urine (c) and faeces (d) were determined by qRT-PCR after immunization.

**
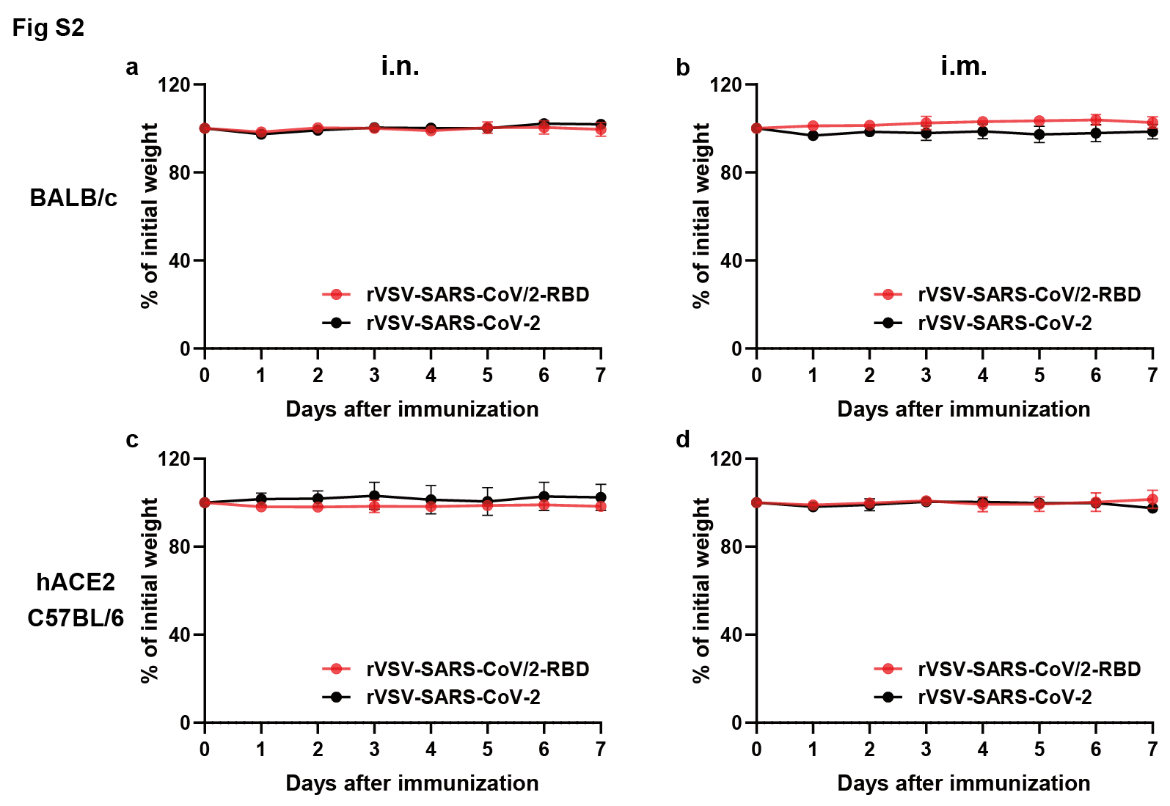
**

**Figure. S2.** **Weight changes of mice after immunization. (a–b)** Groups of female BALB/c mice (n = 6) or **(c–d)** female hACE2 knock-in C57BL/6 mice (n = 4) were immunized with a single dose of rVSV-SARS-CoV-2 (black) or rVSV-SARS-CoV/2-RBD (red) via **(a, c)** i.n. (10^5^ FFU/animal) or **(b, d)** i.m. route (4 × 10^5^ FFU/animal). Following vaccination, the body weight was monitored for 7 d.


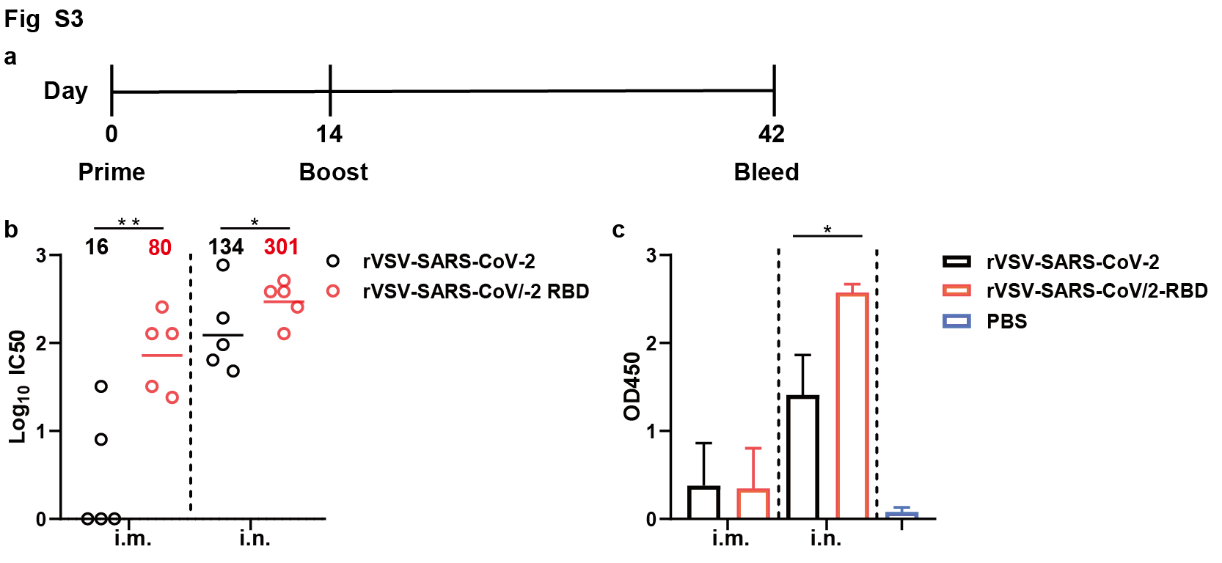


**Figure. S3.** **Immunogenicity of rVSV-SARS-CoV-2 and rVSV-SARS-CoV/2-RBD in golden Syrian hamsters**. **(a–c)** Groups of golden Syrian hamsters (n=5) were immunized with rVSV-SARS-CoV-2 (black) or rVSV-SARS-CoV/2-RBD (red) via i.n. (10^5^ FFU/animal) or i.m. route (10^5^ FFU/animal) at day 0 and 14. **(a)** Scheme of vaccination. **(b)** The NAb titers were determined by live SARS-CoV-2 neutralization assay at 28 d post second immunization. **(c)** SARS-CoV-2 S RBD specific IgG responses in the sera were determined by ELISA (1000 × dilution). Statistical significance was determined using unpaired two-tailed student’s *t* test. ***p* < 0.01, **p*<0.05. Error bars indicate standard deviation of the mean.
